# The relation of age to the severity of Type I diabetes in children

**DOI:** 10.4103/1319-1683.71990

**Published:** 2010

**Authors:** Suliman H. Al-Fifi

**Affiliations:** *College of Medicine, King Khalid University, Abha, Kingdom of Saudi Arabia*

**Keywords:** Type 1 diabetes, children, severity, biochemical, laboratory

## Abstract

**Objective:**

To study the relationship between the age and severity of Type 1 diabetes in children 0 - 5 years and more than 5 years of age admitted to Aseer Central Hospital, Southwestern Saudi Arabia over a 7-year period.

**Materials and Methods:**

A retrospective review of children less than 13 years of age with Type 1 diabetes admitted to the Pediatric Department, between 1st January 2000 to 31st December 2006.

**Results:**

A total of 181 children with Type1 diabetes were admitted to the hospital during this period. Of these, 27.6% were children 5 years or less, while 72.4% were more than 5 years of age. The duration of symptoms was longer in younger children compared to older patients. Diabetic ketoacidosis was present in 31.4% of the younger children, and in 15.3% of the children more than 5 years old. Hospital stay was also longer in children less than 5 years of age. Most significant differences were in the younger children’s group and affected the biochemical test results.

**Conclusion:**

The present study showed that more younger children present to the hospital late, and in a state of diabetic ketoacidosis compared to older patients. Efforts should be directed at improving the knowledge and skills of the primary health care personnel to be able to diagnose and refer these cases earlier.

## INTRODUCTION

Type 1 diabetes mellitus (T1DM) is one of the most common chronic diseases in children. It is caused by absolute insulin deficiency following the destruction of the insulin-producing pancreatic beta cells. Most commonly, it presents in childhood, but one-fourth of all cases are diagnosed in adults. Type 1 diabetes accounts for approximately two-thirds of the newly diagnosed cases in patients ≥19 years of age.[1–3]

Type 1diabetes has a bimodal distribution with one peak at 4 to 6 years of age and a second at early puberty (10 to 14 years of age).[4–6]

The period between the onset of symptoms and the diagnosis of the disease is variable. This can be as long as a few weeks to months when the level of education of the family is low or health facilities are inadequate. When there is a family history of Type1diabetes, this period can be as Short as a few days if the parents are very observant. The other important factor is the age of the patient. Younger children are likely to present with vague symptoms such as apathy, irritability, restlessness while older children usually present with the classical symptoms of polyuria, polydipsia and weight loss. The diagnosis in girls is also likely to be more delayed than boys for no clear reason.[7,8]

Younger children are also likely to present critically ill and this is reflected by the higher rates of diabetes ketoacidosis (DKA) in them compared to older children. A more severe degree of dehydration is also encountered in this age group. This is because they have a higher rate of respiratory and gastrointestinal infections which may delay the diagnosis. In these children the metabolic derangements may also take longer to correct.[8–14]

The prevalence of DKA decreased with age from 37.3% in children aged 0 to 4 years to 14.7% in those aged 15 to 19 years.[15]

The frequency of DKA at the onset of the disease correlates inversely with the incidence of Type 1 diabetes and is more common if there is negative family history, poor socioeconomic conditions, less desirable health insurance coverage, and lower parental education.[10] In a local study done in the northwest of Saudi Arabia, DKA was diagnosed in 55.3% at the onset of the disease and girls were found to present in DKA more frequently than boys, with a ratio of 1.4:1.[16]

This study was conducted to compare the clinical and laboratory characteristics of diabetic children of two different age groups admitted to our hospital with a special interest in their presentation and their hospital course.

## MATERIALS AND METHODS

All children aged 0-13 years of age with Type 1 diabetes admitted to the pediatric department of Aseer Central hospital, Southwestern Saudi Arabia, from 1^st^ January 2000 to 31^st^ December 2006 were included in the study. Any patient older than 13 years of age or admitted for any reason other than diabetes was excluded.

Their files were analyzed to collect the following data: name, age, sex, file number, clinical presentation at the time of admission to the pediatric ward or to the pediatric intensive care unit (PICU). Height, weight, and weight loss were determined. The mental status was registered as affected or not as well as their initial biochemical tests results. A child with diabetic ketoacidosis was defined as any child presenting with pH < 7.3, bicarbonate < 15 and the presence of urinary ketones. The duration of hospitalization was also noted.

The study was approved by the chairman of the research committee at our institution. Aseer central hospital is the only tertiary hospital in the region which receives all the complicated cases diagnosed at the primary health care centers and other secondary hospitals.

### Statistical analysis

The collected data were then categorized into those children 0-5 years of age and of children more than 5 years age. Statistical analysis was then undertaken to compare the two groups using two tailed Student’s t test and Chi square tests wherever indicated with significance level at *P* <0.05.

## RESULTS

Table 1 describes the comparison of the clinical presentation of the two groups of children. It shows that the mean age of the older group was 9.6 ± 1.7 years while the mean age for the younger children was 2.3 ± 1.3 years. The duration of symptoms before admission was significantly longer (19. 3 ± 10.1 days) in the younger children than in the older children (7.8 ± 13.3 days). Moreover, the younger children also had a significantly longer hospital stay (8.2 ± 9.5 days) compared to 4.6 ± 3.5 days for the older group. There was also differences in the clinical picture at the time of admission between the two groups. Thus, a significantly higher percentage of the younger children presented with accompanying upper respiratory tract infections (URTI) and dehydration (30% and 72% versus 10.7% and 47.3% respectively). However, abdominal pain was significantly more frequent in the older children than the younger children (61.1% versus 26%). A significantly higher percentage of the younger children (31.4%) than the older group (15.3%) had ketosis and ketone bodies in their urine at the time of admission (31.4%).

**Table 1:** Clinical presentation of both groups of children at the time of admission

Clinical Presentation	Group <5 years N = 50 ± S.D. X	Group >5 years N = 131 ± S.D. X	*P* value
Age (years)	2.3 ± 1.3	9.6 ± 1.7	
Duration of symptoms (days)	19.3 ± 10.1	7.8 ± 13.3	0.05
Hospital stay (days)	8.2 ± 9.6	4.6 ± 3.5	0.012
Weight loss (kg)	1.72 ± 0.54	1.57 ± 0.40	0.04
	N (%)	N (%)	
Onset with ketosis	16 (31.4%)	20 (15.3%)	0.012
URTI	15 (30.0%)	14 (10.75)	0.003
Abdominal pain	13 (26.0%)	80 (61.1%)	0.001
Polyuria/polydypsia	41 (80.3%)	95 (72.5%)	0.31
Dehydration	36 (72.0%)	62 (47.3%)	0.004
Mental status affection	23 (46.0%)	75 (57.3%)	0.18

URTI: upper respiratory tract infections

Table 2 depicts the comparison of biochemical tests results between the two groups of children. It shows that, although the random blood sugar RBS was not significantly different between both groups, the HbA1c was significantly lower in the younger group of children with a mean of 8.8% ± 2.6% compared to 11.2 % ± 2.2% for the older group.

**Table 2:** Comparison of biochemical tests results between both groups of children at the time of admission

Clinical Presentation	Group <5 years N = 50 ± S.D. X	Group >5 years N = 131 ± S.D. X	*P* value
RBS (mg/dl)	428.8 ± 201.9	454.2 ± 202.5	0.46
HbA_1_c (%)	8.8 ± 2.6	11.2 ± 2.2	0.001
pH	7.2 ± 0.11	7.3 ± 0.15	0.04
ABE	−13.9 ± 7.3	−9.8 ± 6.4	0.001
HCO_3_	9.9 ± 6.3	14.2 ± 11.3	0.01

RBS: random blood sugar, HbA1c: glycosylated hemoglobin, pH: acid status, ABE: acid base excess, HCO_3_: bicarbonate

The blood pH was significantly more acidic in the younger children (7.2 ± 0.11) compared to 7.3 ± 0.15 in the older children. Consequently, the ABE was significantly higher in the younger children (-13.9 ± 7.3) compared to (-9.8 ± 6.4) in older patients. The level of serum bicarbonate was a significantly lower in the younger children.

## DISCUSSION

With the improvement in medical care, Type 1 diabetes can be easily recognized if it is considered by the physician during the assessment of a sick child. The availability of biochemical testing will help the primary care provider to determine the severity of the disease and direct the management of the patient. However, since very young child do not always show clear symptoms, more time has to be be spent to elicit the important information from parents or caregivers. This approach will help to make the diagnosis before any serious metabolic derangements take place.

Previous studies from different areas of Saudi Arabia reported higher rates of DKA at the onset of the disease ranging from 55-77% compared to our study (46.7%).[16]

The lower rates of DKA noticed in the current study compared to the previous reports may be due to better awareness of the disease by the community and easy access to health care.

In the present study, it was found that younger children presented later to the hospital than older children. This might be due to the absence – in most cases – of the classical symptoms of polyuria, polydipsia and weight loss noticeable in older children. Moreover, abdominal pain was less apparent in young children than older ones. This delay contributed to the severity of the disease in this group as shown by the higher frequency of DKA compared to older children (31.4% compared to 15.3) respectively.

It is to be noted here that, despite presenting a more severe disease the younger children had a significantly lower HbA1c% denoting a shorter period of uncontrolled glycemia, compared to the older children, thus compounding the insidious onset of DKA in younger children

It is well known that T1DM in children, often presents with ketosis as the metabolic changes are rapid in this type of diabetes.[17] In the present study, almost a third (31.4%) of the younger patients presented with ketosis, which was double that of the older children (15.3%).

The lack of awareness of the parents as well as the nonspecific early signs and symptoms might be responsible for this discrepancy between the two groups.[7,8] The longer duration of signs and symptoms as well as the profound metabolic disturbances, and the rapid loss of more fluid by young children resulted in the significantly higher number of young children who showed signs of dehydration and ketoacidosis.[18]

URT infections are known to be one of the triggers of ketoacidosis in diabetic patients.[19] It was a significant presenting finding in the younger children compared to the older group in our study. This might sometimes mask and delay the diagnosis especially at primary health care centers. The biochemical test results show that the older children had a significantly higher RBS. However, both figures, in fact, are so high that the difference between them is of little consequence even if statistically significant.

It has been also noted that with the onset of dehydration in diabetic children, the glumerular filtration rate (GFR) decreases resulting in decreased clearance of glucose and ketones from the circulation. This increases the likelihood of DKA.[20] The acidosis, however, as witnessed by the significantly lower pH and higher ABE in the young children especially when compounded by a significantly higher degree of dehydration carries a special risk of serious complications.

### Recommendations

Although this study has a limitation of not including all cases in the region, as some were treated in other institutions, it can be considered an indicator of the clinical picture of children affected with T1DM.

Early recognition of the condition in young children should be sought by improving the awareness of the community as well as the primary care physicians to the possibility of T1DM in the high risk group especially after prolonged vague symptoms of infection. For known diabetics, an early biochemical testing might be of help towards early diagnosis.
